# Resting State Functional Connectivity in Perfusion Imaging: Correlation Maps with BOLD Connectivity and Resting State Perfusion

**DOI:** 10.1371/journal.pone.0027050

**Published:** 2011-11-04

**Authors:** Roberto Viviani, Irene Messina, Martin Walter

**Affiliations:** 1 Department of Psychiatry and Psychotherapy III, University of Ulm, Ulm, Germany; 2 Department of Applied Psychology, University of Padua, Padua, Italy; 3 Clinical Affective Neuroimaging Laboratory, Department of Psychiatry, Otto von Guericke University, Magdeburg, Germany; Institute of Psychology, Chinese Academy of Sciences, China

## Abstract

Functional connectivity is a property of the resting state that may provide biomarkers of brain function and individual differences. Classically, connectivity is estimated as the temporal correlation of spontaneous fluctuations of BOLD signal. We investigated differences in connectivity estimated from the BOLD and CBF signal present in volumes acquired with arterial spin labeling technique in a large sample (*N* = 265) of healthy individuals. Positive connectivity was observable in both BOLD and CBF signal, and was present in the CBF signal also at frequencies lower than 0.009 Hz, here investigated for the first time. Negative connectivity was more variable. The validity of positive connectivity was confirmed by the existence of correlation across individuals in its intensity estimated from the BOLD and CBF signal. In contrast, there was little or no correlation across individuals between intensity of connectivity and mean perfusion levels, suggesting that these two biomarkers correspond to distinct sources of individual differences.

## Introduction

The investigation of functional connectivity in the resting state has recently emerged as a powerful approach to characterize the intrinsic functional architecture of the brain [1]–[3]. The comprehensive map of functional connections in the resting brain (‘functional connectome’) is increasingly seen as a property of the brain activity in the unconstrained resting state [4]–[5], identified by spontaneous low-frequency fluctuations of the fMRI signal (<0.1 Hz) occurring in temporal synchrony across distinct large-scale brain networks [6]. The potential importance of functional connectivity is highlighted by its role in the identification of such diverse biomarkers of brain function as the effect of pharmacological agents [7]–[9], the genetics of individual differences [10], mental disorders [11]–[12], functional brain anatomy [13]–[16] and development [17]–[21].

Current knowledge of the functional connectome in man is based on images acquired with standard BOLD-EPI techniques exploiting the magnetic susceptibility properties of oxygenated blood [2], [22]. The purpose of the present study is the description of the functional connectome using perfusion imaging, a non-invasive magnetic resonance technique that allows quantitative estimates of perfusion levels (arterial spin labeling, ASL, [23]). Several pilot studies of methodological character conducted in diverse laboratories [24]–[27] have demonstrated the validity of connectivity maps obtained with perfusion imaging techniques. Although the signal-to-noise ratio of perfusion imaging is lower than in standard BOLD-EPI, the spatial patterns of low-frequency fluctuations in the resting state obtained with perfusion imaging appear to correspond most closely with those obtained with estimates of oxygen consumption [28]. Furthermore, perfusion imaging provides information not available in standard BOLD-EPI. Using perfusion imaging it is possible to investigate not only connectivity networks, but also absolute levels of perfusion [27]. Because studies of mean level of perfusion at rest were at the origin of the concept of default network arising from PET studies [5], perfusion imaging potentially integrates older and newer approaches to the study of the resting state in a single technique. Both aspects of the resting state, mean activity levels and functional connectivity, are available as potential markers of individual differences in functional brain architecture, and represent a promising current development in the study of human brain function. Functional connectivity estimated from ASL sequences may provide the benefit of directly assessing mean CBF changes and changes of connectivity in studies where both aspects of brain function need to be accounted for.

Notwithstanding these promising initial results, no study has systematically described the functional connectome with perfusion imaging techniques. Here we provide the description of the main indices of connectivity in a well-characterized sample of *N* = 265 young healthy human volunteers [29]–[30], in which perfusion images at rest were acquired with a continuous ASL technique [31]–[33].

Our study aimed at addressing three issues. The first was the concordance of connectivity maps estimated on the CBF and BOLD parts of the signal, extending reports in the literature on the possibility of estimating connectivity from ASL data with the analysis of a much larger, representative sample. If CBF and BOLD provide estimates of the same connectivity, we would expect not only that the respective maps be similar, but also individual differences in the voxelwise intensity of connectivity to correlate. The second was the existence of connectivity at very low frequencies (below 110 sec). These frequencies cannot be investigated with standard EPI-BOLD techniques due to low frequency confounds such as scanner drift [34], but are available in the CBF signal [35]. The third was whether individuals with high connectivity also display systematically higher or lower baseline perfusion levels. This aspect of the relationship between baseline tone and connectivity cannot be assessed with standard EPI-BOLD techniques, because these latter give no quantitative estimate of baseline activation. The study of baseline brain perfusion or metabolism constitutes one of the earliest empirical approaches to the neuroimaging of mental disorders [36]. Hence, data on an association between baseline perfusion and connectivity inform on the possible existence of a relationship between individual differences detected with these two methodologies.

## Materials and Methods

The study protocol was approved by the ethical committee of the University of Ulm and was in compliance with national legislation, the principles expressed in the Declaration of Helsinki, and the Code of Ethical Principles for Medical Research Involving Human Subjects of the World Medical Association. All participants gave written informed consent.

Participants were recruited from local schools and university and by local announcements. Exclusion criteria were neurological or medical conditions, use of medication, or a history of mental illness, and subclinical structural abnormalities. The sample comprised 265 individuals (143 females) aged between 18 and 46 years at the time of the scan (mean age 24.3, std. dev. 4.5).

All magnetic resonance imaging (MRI) data were obtained with a 3-Tesla Magnetom Allegra (Siemens, Erlangen, Germany) MRI system equipped with a head volume coil. All participants were scanned at the Department of Psychiatry and Psychotherapy III of the University of Ulm. A continuous arterial spin-labeling technique was used as described in ref. [33]. This technique relies of the effect of an inversion pulse, applied selectively at the neck to perturb the spins of protons in water molecules in transit with arterial blood. When these protons subsequently diffuse in brain parenchyma, they are less susceptible to imaging due to the perturbed spins. Estimates of perfusion are made by subtracting these images from images acquired without labeling. Interleaved images with and without labeling were acquired for 8 min (120 acquisitions) by using a gradient-echo echo-planar imaging sequence (TR/TE 4000/17, flip angle 90°, bandwidth of 3005 Hz/Pixel, field of view of 22 cm, image size 64×64×15 voxels, slice thickness 6 mm with a gap of 1.5 mm, giving a voxel size of 3.44×3.44×7.50 mm). A delay of 1 sec was inserted between the end of the labeling pulse of 2 sec and image acquisition to reduce transit artefacts. The SPM5 package was used (Wellcome Department of Cognitive Neurology, London; online at http://www.fil.ion.ucl.ac.uk) for realignment and stereotactic normalization to an EPI template (Montreal Neurological Institute, resampling size: 2×2×2 mm). After realignment, data were residualized relative to the realignment parameters. To ensure that CBF values were not contaminated by the BOLD signal, the dataset was split into a high-pass filtered series with cut-off 12.5 sec [25], and a low-pass series obtained as the residuals of the filtering. Filtering was carried out using SPM functions, which implement it as a projection of the data in the subspace spanned by the appropriate basis functions of a discrete cosine transform. The high-pass filtered series was used to obtain the CBF signal, while the low-pass filtered series contained the EPI-BOLD signal. While aimed at avoiding contamination of CBF signal from BOLD signal, this preprocessing step also has the effect that the BOLD and CBF analyses were conducted on two sets of uncorrelated data under the null (because the subspaces of the basis function and their residuals are orthogonal), allowing unbiased verification of concordance in the estimated connectivity maps. Reconstruction of rCBF values was obtained using software implementing eq. (1) of ref. [33] and the ‘simple subtraction’ method [37] on the high-pass filtered series. Note that after reconstruction of the CBF signal, the previous high-pass filtering step does not imply that the reconstructed signal be restricted to the high frequency band of the cutoff, because the CBF signal arises from differences from subsequent scans (for details of the spectral analysis of the subtracted series, see ref. [25]). All volumes were smoothed using an isotropic Gaussian kernel of full width half-maximum (FWHM) 6 mm. A volume mask was obtained by thresholding *a priori* tissue probability maps provided by the SPM package at 0.7 for brain. Because of individual differences in coverage, slices above +70 mm were excluded from analysis (the spin inversion giving rise to the perfusion signal decays rapidly as the EPI ascending sequence progresses). The cerebellar region was also excluded, because affected by large variance in our data (these slices are close to where the inversion pulse is given).

Connectivity was given by seed-based Pearson correlation analysis ([1]; for a useful review of theory and methods, see [34], [38]) based on six well-established seed regions [2], [4]. Three of these commonly exhibit activity increases during the execution of demanding tasks: the intraparietal sulcus (IPS, Montreal Neurological Coordinates *x*, *y*, *z*: −25, −57, −46), the right frontal eye field (FEF, 25, −13, 50), and the middle temporal region (MT, −45, −69, −2), while the other three are normally deactivated: ventromedial prefrontal cortex (ventral anterior cingulate, vACC, −1, 47, −4), the posterior cingulated/precuneus (PCC, −5, −49, 40), and the lateral parietal cortex (LP, −45, −67, 36). We chose these single voxel seeds primarily for comparability with the existing literature. A recent study showed that more elaborate seed choices lead to similar results [39]. A voxelwise regression on the seed voxel was computed after standardizing the signal from all voxels, including the seed voxel. Estimates of connectivity in the literature based on the BOLD signal band-pass filter this signal at cutoffs around 12 sec and 110–120 sec to avoid the confound of the slow drift of the signal present in EPI-BOLD images [38]. To ensure comparability, we applied a band-pass filter (12.5 sec, 0.08 Hertz, and 111 sec, 0.009 Hertz) to both the BOLD and the CBF series prior to computing connectivity, using the same filtering and residual-taking approach as previously. The low-pass filtered signal in the CBF images (hence containing frequencies lower than 0.009 Hertz) was used to estimate low-frequency connectivity. Hence, the CBF connectivity analyses of the band-pass and low-pass filtered signal were conducted on two sets of uncorrelated data, allowing unbiased verification of concordance in the estimated connectivity maps.

To control for spatial correlation due to physiological noise [40]–[41], which may spuriously increase positive correlations [42], a set of seeds obtained empirically from a set of 35 time-of-flight images were included as nuisance covariates in all connectivity estimates [29]. These seeds controlled for the effect of the internal carotid artery and the basilar artery. In addition, time series from four additional seeds in CSF and white matter (as in ref. [4]) were added as nuisance covariates to adjust for confounding sources of regionally-specific noise. CSF seeds were obtained for the posterior arm of the lateral ventricles (two spheres of radius ∼5 mm centered at coordinates ±23, −45, 9), the anterior arm (ellipsoids of axis lengths ∼1, 5, 5 mm centered at ±2, 13, 6) the posterior cisterna (sphere of radius ∼5 mm centered at 0, −41, −1). The white matter time series were extracted from two ellipsoids of size ∼6, 15, 6 mm centered at coordinates ±25, −10, 35. The global signal of each volume and a linear trend were added as confounding covariates to the model. All covariates, including the signal from the seeds, were centered. Note that this ensures that connectivity and mean perfusion estimates were conducted on two sets of uncorrelated data, allowing unbiased estimation of correlation between connectivity and mean perfusion values.

Interindividual correlation maps were obtained by computing voxelwise correlations at the second level between two sets of connectivity maps, provided by each of the 265 participants. For example, to compute the interindividual correlation between CBF and BOLD connectivities, the individual connectivity maps from one modality separately provided in each voxel 265 datapoints (one from each participant) that were correlated with the datapoints from the same voxel in the other modality. Likewise, to compute the interindividual correlation between mean CBF levels and CBF connectivity, the rest perfusion map in each participant (averaged in each voxel from the 8 min of scanning) separately provided in each voxels the datapoints that were correlated with the datapoints in the same voxel in the connectivity maps. This procedure provides a measure of voxelwise concordance of individual differences in connectivity intensity, or of the voxelwise concordance between individual connectivity and rest perfusion levels, and follows standard neuroimaging approaches to model fitting, except that the model changed in each voxel.

The coefficients of the fit of the seed variable on the data were brought to the second level to model individual effects as a random factor, and significance values were estimated with a permutation method [43] using 8000 permutations. The significance of correlation maps was computed analogously, making provisions for the voxelwise model. Images were generated with the software Caret version 5.62 [44]. Peak-level corrected significance levels in images were set at the threshold *p* = 0.05.

## Results

**Figure 1:**
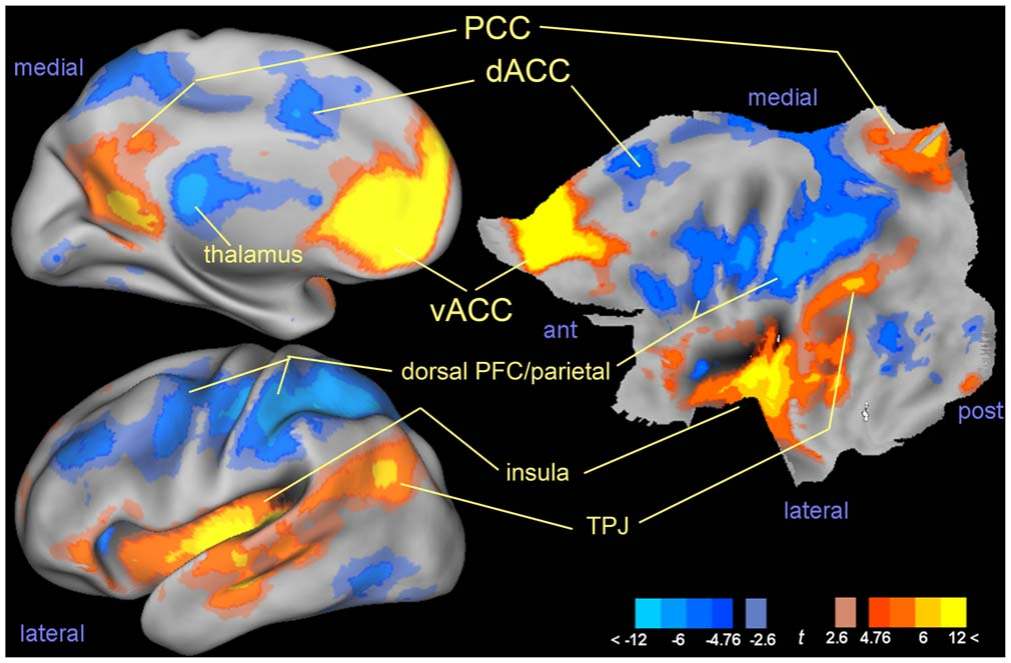
Maps of *t* values illustrating the connectivity between the seed in the left vACC and the cortical voxels in the left hemisphere. In orange/yellow positive correlation levels; in blue, negative correlations. Maps were thresholded at both the uncorrected *p* = 0.005 level and shown in shaded color; full colors were used for effect significant at the more stringent peak-level *p* = 0.05 FWE-corrected threshold (*t* levels of 4.7–4.8). On the left, *t* maps were projected on an inflated rendering of the cortex for both the medial (top) and lateral (bottom) aspects of the left hemisphere. On the right, the same rendering on a flat rendering of the whole hemisphere. One can see that the superior third of flattened representation corresponds to the medial cortical surface, while the rest corresponds to the lateral surface. The main anatomical structures are indicated to illustrate reading of the flat rendering. Abbreviations: PCC: posterior cingulus; dACC: dorsal anterior cingulus (medial prefrontal cortex); vACC: ventral anterior cingulus (medial prefrontal cortex); PFC: prefrontal cortex; TPJ: temporo-parietal junction; ant, post: anterior, posterior.

Figure 1 shows maps of connectivity levels in perfusion data obtained in the left hemisphere from the vACC seed. One can see robust positive connectivity medially in the posterior cingulus (PCC), and laterally in the insular cortex reaching posteriorly to the temporo-parietal junction. The main purpose of this figure is familiarizing with the flattened representation shown on the right (we use this representation in the rest of the study to compactly display both medial and lateral aspects of a hemisphere). The same conventions are used to display effects in the whole study; significant results, corrected at peak-level, are in full intensity colors.

### Connectivity maps from CBF and BOLD data

**Figure 2:**
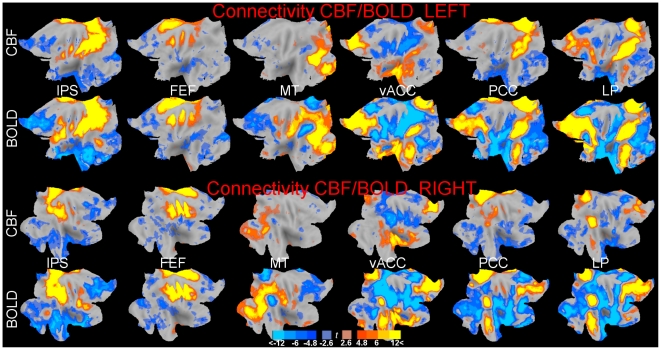
Maps of connectivity between the seed voxels in the IPS, FEF, MT, vACC, PCC, and LP regions (from left to right) in the left and right hemisphere (top and bottom half of image). For each hemisphere, CBF and BOLD connectivity are shown (upper and lower row). The maps were rendered on the flattened cortex and thresholded at both the uncorrected *p* = 0.005 level and shown in shaded color; full colors were used for effect significant at the more stringent peak-level *p* = 0.05 FWE-corrected threshold.

Figure 2 shows connectivity maps obtained from CBF and BOLD data. The connectivity of seeds located in areas activated by focused tasks (first three columns) shows the known recruitment of dorsal areas [4], while seeds located in the default system network recruited ventral areas in both the medial and lateral aspects of the hemispheres. Connectivity also displayed the known rough symmetry between left and right hemispheres [2], although it was stronger in the left due to the left location of seeds (with the exception of the FEF seed). One can see the qualitative similarity of these maps; however, it is apparent that CBF connectivity was less intense and extensive than BOLD connectivity. A further difference emerging from this Figure is the apparent tendency of BOLD connectivity to be more extensive in the negative direction in the vACC, PCC, and LP seeds. Because of the similarity of left and right connectivity, we focus for brevity on the left hemisphere in the rest of the study.

**Figure 3:**
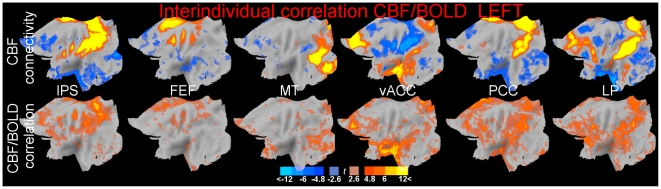
Connectivity originally measured in the CBF signal (top row) and its individual correlation with the connectivity measured in the BOLD signal (bottom row). Maps of connectivity between the seed voxels in the IPS, FEF, MT, vACC, PCC, and LP regions (from left to right) in the left hemisphere. Thresholds and conventions as in Figure 2 (full intensity colours correspond to peak-level corrected significance).

CBF and BOLD connectivities were also correlated across individuals (individuals with higher BOLD connectivity also had higher CBF connectivity, and vice versa). Figure 3 shows the CBF connectivity for reference in the top row, and in the bottom row its correlation with BOLD connectivity, computed voxel by voxel. Three aspects of these maps are worthy of note. First, correlations across the volume were only positive. Even at uncorrected threshold levels, there were few or no voxels where the correlation was negative. Second, formal testing resulted in significant positive correlations for all seeds. Third, the significant positive correlation was specifically localized in the areas of positive connectivity. In contrast, negative connectivity elicited much less interindividual correlation between BOLD and CBF.

### CBF connectivity at very low frequencies

**Figure 4:**
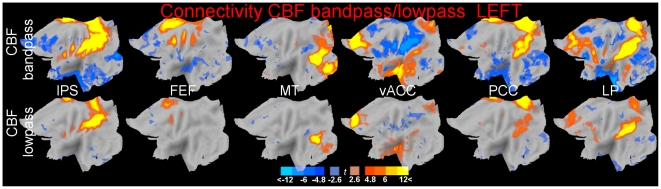
Connectivity in the original band-passed CBF signal (top row) and residual low-passed signal (bottom row). Maps of connectivity between the seed voxels in the IPS, FEF, MT, vACC, PCC, and LP regions (from left to right) in the left hemisphere. Thresholds and conventions as in Figure 2 (full intensity colours correspond to peak-level corrected significance).

Figure 4 shows maps of CBF connectivity at frequencies below 0.009 Hz (111 sec). As in the previous figure, in the top row CBF connectivity from the band-passed signal used in the main analysis is displayed in the top row for comparison. These maps show the existence of connectivity at these very low frequencies, spatially distributed as in the band-passed signal. However, the intensity of the connectivity was lower. Negative connectivity, which was less intense in the band-passed connectivity maps, was almost absent.

### Correlation between connectivity and baseline perfusion

Tests of voxel by voxel correlation between connectivity and baseline perfusion failed to reach significance at peak-level corrected levels in all seeds. Cluster-level corrected significance was reached for a positive correlation between baseline perfusion and MT connectivity in the left calcarine cortex (*x*, *y*, *z*: 14, −52, 6, BA18, *k* = 622, *p* = 0.01), and for a negative correlation between the PCC seed and the surrounding cingular region (*x*, *y*, *z*: −16, −38, 28, BA23/26, *k* = 2565, *p*<0.001).

## Discussion

This study provided empirical evidence in a large sample of the suitability of arterial spin labeling data for the investigation of connectivity. It also provided evidence of the broadband character of connectivity, and of the distinct information provided by connectivity and mean perfusion levels at rest. We discuss each of these issues in turn.

Connectivity maps from CBF and BOLD signal were regionally similar. The main difference between the CBF and BOLD estimates of connectivity of this study was the quantitatively lower intensity of CBF connectivity. Two aspects of the perfusion signal may explain this observation. First, relative to BOLD, the signal-to-noise ratio of CBF signal is lower [37]. When affecting the signal both in the seed voxel and in the rest of the volume, lower signal-to-noise ratios may result in lower connectivity estimates. Second, because two volumes are required to produce a CBF image, there are about twice as much data for the estimate of BOLD connectivity than CBF. This is another reason why connectivity, as measured by *t* maps or significance levels, was greater for BOLD than CBF data.

A second aspect deserving comment is the larger extension of negative connectivity in BOLD images, especially visible in the seeds from the default network system. As previously noted [45], since connectivity is computed relative to mean signal levels to avoid global signal oscillations to appear as positive connectivity, areas with little or no connectivity may artefactually appear as negative (see however [42], [46]). In general, the spatial distribution of positive connectivity was more replicable across frequencies and techniques, even if at different intensities.

Notwithstanding these differences, two findings spoke for the qualitative equivalence of BOLD and CBF connectivities. The first was their almost identical spatial distribution, notwithstanding the different intensities. The second was the existence of a correlation of BOLD and CBF connectivity across subjects. This correlation was present only in areas in which connectivity was present, especially positive connectivity, indicating the specificity of the concordance between the BOLD and CBF data. As a whole, the correlation across subjects suggests that perfusion imaging may be used in studies of individual differences in connectivity.

A further result of this study was the presence of connectivity at very low frequencies (<0.009 Hz), with a spatial distribution similar to the connectivity at higher frequencies. This result shows that connectivity is present at a wide band of frequencies, adding to previous findings of its extension to the high-frequency spectrum [47]. This result also shows that in studies of connectivity with perfusion methods it is not necessary to high-pass the signal, thus increasing the amount of data available in the estimate.

The final result of this study was that very little, if any, associations between high connectivity and high or low mean perfusion levels across subjects were found. This conclusion is made plausible by two observations. First, this finding contrasted with the very large and significant correlations found when comparing connectivities obtained from BOLD and CBF signal, which attested the presence of connectivity signal in the CBF connectivity maps. The validity of the rest perfusion signal is documented in previous analyses of this dataset [29]. Second, the null finding in a large sample such as the one of this study implies that only small correlations would escape detection. This result suggests that studies of individual differences in connectivity do not replace studies of mean perfusion. These studies, therefore, potentially provide information about two distinct forms of differences between individuals.
